# Prevalence and Factors Associated with Hepatitis C Virus Seropositivity in Female Individuals in Islamabad, Pakistan

**Published:** 2010

**Authors:** Anjum Hashmi, Khalid Saleem, Jamil Ahmed Soomro

**Affiliations:** 1 Community Health Officer Department of Community Health Prf Medical Center Karachi Pakistan; 2 Medical Superintendent, PINS Complex Hospital Nalore, Islamabad, Pakistan; 3 NMCH Officer WHO, Pakistan

**Keywords:** Hepatitis C, Risk factors, Prevalence, Pakistan

## Abstract

**Objectives:**

An estimated 150-200 million people worldwide are infected with hepatitis C. Only limited information about the epidemiology of Hepatitis C virus (HCV) infection is available. The aim of this study was to determine the prevalence of anti-HCV antibodies and the possible factors for transmission in the female population of a largely urban city, Islamabad, Pakistan.

**Methods:**

A cross-sectional study was conducted from May to August 2006 in Islamabad. The city is divided into forty union councils. Five union councils were selected randomly and then, we randomly selected 252 female households (n = 252) of age ranges between 15-50 years who were able to read and write the self-administered questionnaires. Those with severe debilitating disease, physical or mental handicapped or those who did not give consent and known cases of HCV were excluded. The primary outcome variables were HCV seropositivity and factors as history of major surgical procedure, blood transfusion and intravenous drug use.

**Results:**

The mean age of participants was 33.21 (9.95) years and HCV seropositivity prevalence was 62 (24.6%). Final forward stepwise multiple logistic regression showed blood transfusion [OR, 10.09; 95% CI: 1.95-52.25], dental procedure [OR, 5.38; 95% CI: 2.31-12.50] and dilation and curettage [OR, 3.86; 95% CI: 1.86-8.01] were significantly associated with HCV seropositivity.

**Conclusions:**

The study highlights the poor quality of care provided and a massive need to educate general population including patients as well as health professionals and allied health workers for controlling, combating and preventing the wild epidemic of HCV.

## INTRODUCTION

Hepatitis C is a blood-borne infectious disease caused by the hepatitis C virus (HCV), affecting the liver. An estimated 150-200 million people worldwide are infected with hepatitis C. Prevalence is higher in some countries in Asia and Africa. Egypt has the highest seroprevalence for HCV up to 20% in some areas.^1^ Hepatitis C infects nearly 200 million people worldwide and 4 million in the United States. The factors associated with HCV are intravenous drug use (p < 0.001), blood transfusion (p < 0.01), tattoos (p < 0.001), previous hospitalization (p < 0.05), history of sexually transmitted disease (STD) (p < 0.001), and lack of travels outside of Europe (p < 0.05).^2^

Only limited information about the epidemiology of HCV infection especially in females is available in high prevalence areas like Pakistan. Previous study has suggested a prevalence of 9% HCV seropositivity among a sample of patients in Mardan, Pakistan.^3^ Although HCV infection has been identified as one of the major causes of chronic hepatitis CLD and hepatocellular carcinoma its prevalence in the female population and associated factors like minor surgical procedures, biopsy, endoscopy, and dilation and curettage (D & C) are largely unknown.

The aim of this study was to determine the prevalence of anti-HCV antibodies and the possible risk factors for transmission in the female population of a largely urban based city, Islamabad.

## METHODS

The study was conducted in Islamabad, capital of Pakistan having a population of around 0.9 million (1997) with population growth rate of 2.6% (1997). Female to male ratio is 94 per 100, meaning our study population was 0.85 million (1997). Currently the approximate population is well above 1.5 million.

The study was carried out from May 2006 to August 2006 in Islamabad, Pakistan. All female inhabitants of age range between15-50 years living in the study site were eligible to participate in the study. A cross-sectional study design was used to achieve the primary objective. To determine the prevalence of HCV seropositivity and evaluate potential risk factors by comparing HCV seropositive and seronegative female individuals. A multi stage sampling was used. The city was divided into 40 union councils. We randomly selected five union councils and then female households were randomly selected among five selected union councils. Our sample size of the study was 252.

Two co-researchers also medical reactionaries registered with Pakistan Medical and Dental Council (PMDC) went to the selected houses and approached the female head in the family. They explained the purpose and objectives of the study and asked for written informed consent from participants before administering the questionnaire and collecting a blood sample. The self administered questionnaires were available in both Urdu and English versions. The questions gathered demographic characteristics including age, socio-economic status based on monthly incomes (classified into high class, upper middle class, lower middle class, and poor), marital status and also number of years in education (classified into primary educated, secondary educated, and graduate), history of surgical procedure, blood or blood products transfusion, history or current use of Intravenous drug use (IVDU), history of tattooing or scarification, history of dental treatment, ear piercing, minor surgical procedures like biopsy and D & C, cesarean section (C-section), abroad visits and working as a health worker (included doctors, nurses, and all laboratory and paramedical staffs etc).

Five ml of blood was collected from each case and was sent to the designed laboratory. Sera were separated by centrifugation and were tested for anti HCV antibodies within one hour. The anti HCV antibodies were tested by the Dot immunochromatographic method. The immunochromatographic test is for HCV core antigen detection. It is easily to performed, ripid, highly sensitive and specific test, based on the immunochromatographic strip. Positive reactions can be detected weakly at a 1:15 dilution of the serum and more strongly in 1:10, 1:5, 1:2 and 1:1 dilutions, by the immunochromatographic strip. In addition, the test was capable of detecting 0.25–12.0 μg of the recombinant protein. This immunochromatographic technique opens new perspectives for the diagnosis of hepatitis C during the early seroconversion phase and for rapid core antigen detection.

Results were analyzed for finding the prevalence of Hepatitis C in the target population.

The data was analyzed using the Statistical Package for Social Sciences (SPSS Inc., Chicago, IL) version 13. Descriptive statistics of socio-demographic and other variables of the sampled population were computed. Means and standard deviations (SD) were calculated for quantitative variables and proportions for categorical variables. Logistic regression analysis was performed to measure the association between dependent and independent variables. Odds ratios (OR) and 95% confidence intervals (CI) were calculated from β coefficients and their standard errors. Associations between independent variables were assessed using chi square and only those with significant association were entered to perform multivariate analysis. A multivariate logistic regression model was employed with HCV antibody status as the dependent variable. P values < 0.05 were considered to be statistically significant.

## RESULTS

The descriptive analysis showed that the mean age of the sample was 33.21 (± 9.95) years with minimum and maximum values of 15 and 50 years respectively. HCV seropositivity was 62 (24.6%). About 148 (58.73%) were poor, 80 (31.7%) middle class and 24 (9.52%) were upper class; married were 225 (89.2%) and unmarried 27 (10.7%); 141 (55.9%) were illiterate, 51 (20.2%) passed primary and 40 (15.8%) secondary schools, and 20 (10.7%) were graduated; 70 (27.7%) chew tobacco, smokers were 190 (75.3%) and 8 (3.1%) have history of traveling abroad (Table 1). The frequency of factors associated with HCV seropositivity were as: blood transfusion 12 (4.8%), sexual 2 (0.8%), surgery 88 (34.9%), IVDU (Intravenous drug user) 15 (5.9%), health worker 4 (1.6%), tattooing 6 (2.4%), dental procedures 154 (61.1%), endoscopy 14 (5.6%), biopsy 4 (1.6%), dilation and curettage 134 (53.2%) and cesarean section 36 (14.3%).

**Table 1:** Demographic characteristics of participants

Demographic characters		n (%)
**Age groups** (years)	15-20	18 (7.1)
	21-25	40 (15.9)
	26-30	58 (23)
	31-35	42 (16.7)
	36-40	36 (14.3)
	41-45	24 (9.5)
	46-50	34 (13.5)
**Socio economic**		148 (58.73)
	Poor	
	Middle Class	80 (31.7)
	Upper Class	24 (9.52)
**Marital status**		
	Married	
	Unmarried	27 (10.7)
**Level of education**		141 (55.9)
	Illiterate	
	Primary	51 (20.2)
	Secondary	40 (19.8)
	Gradate	20 (7.9)
**Addiction**		70 (27.7)
	Tobacco chewing	
	Smoking	50 (19.8)
	IVDU	15 (5.9)
**Traveling abroad**	Secondary	8 (3.1)

IVDU: intravenous drug use

The univariate analysis showed that blood transfusion, sexual contact, surgery, health worker, dental procedure, endoscopy, biopsy, dilation and curettage, and cesarean section were significantly associated with HCV seropositivity (Table 2). Final Forward Stepwise (Wald) multiple logistic regression showed blood transfusion [OR, 10.09; 95% CI: 1.95-52.25], dental procedure [OR, 5.38; 95% CI: 2.31-12.50], and dilation and curettage [OR, 3.86; 95% CI: 1.86-8.01] were significantly associated with HCV seropositivity in females (Table 3).

**Table 2:** Factors associated with seropositivity of Hepatitis C

Risk Factors	Anti Hepatitis Positive n (%)	C Antibody Negative n (%)	P value
Blood transfusion	12(4.8)	240(95.2)	< 0.001
Sexual	2(0.8)	250(99.2)	0.013
Surgery	88(34.9)	164(65.1)	< 0.001
Dental procedures	154(61.1)	98(38.9)	< 0.001
Health worker	4(1.6)	248(98.4)	< 0.001
Endoscopy	14(5.6)	238(94.4)	< 0.001
Biopsy	4(1.6)	248(98.4)	< 0.001
Dilation and curettage	134(53.2)	118(46.8)	< 0.001
C- section	36(14.3)	216(85.7)	0.003

**Table 3:** Factors associated with seropositivity of Hepatitis C identified in multiple logistic regression

Risk Factors	OR	95% CI	P value
Blood transfusion	10.094	3.869	0.006
Dental procedure	5.381	2.31-12.50	<0.001
Dilation and curettage	3.869	1.86-8.01	<0.001

## DISCUSSION

We observed a significantly high prevalence 24.6% of HCV in Islamabad. Egypt has a high prevalence up to 20% in some areas. A survey conducted in California showed prevalence of up to 34% among prison inmates.^4^ A case control study by Irfan et al. in Mardan, Pakistan in 2004, showed much lower prevalence of 4.34%.^5^ A study conducted in 2002 in Sindh, Pakistan also showed low HCV prevalence of 9%.^6^ Even in a hospital based study conducted in community clinic of Islamabad in 2004, only 5.31% individuals were positive for anti-HCV.^7^

Studies by Khan et al.^3^ and Muhammad et al.^8^ conducted in Pakistan has showed the history of reused syringes, blood transfusion, dental procedure, surgical operation and tattooing as significant risks. Similarly, Batash et al. also showed intramuscular injections (odds ratio 9.1; 95% CI: 2.0-42.4) and blood transfusions (odds ratio 3.2; 95% CI: 1.2-9.0) were significantly associated with HCV seropositivity.^9^ Our results again reiterate their findings as blood transfusion [OR, 10.09; 95% CI: 1.95-52.25] and dental procedure [OR, 5.38; 95% CI: 2.31-12.50] to be significantly associated with HCV.

Consistent with studies by Cacoub et al.,^10^ transfusion of blood products (21.7 vs. 5.5%; p < 0.0001), and dental treatment (55% vs8.3%; p < 0.0001) and Nafeh et al. previous blood transfusion (10.5; 4.7-23.2) and sexual contact with an intravenous drug user (6.9; 3.1-15.2). Delage et al.^12^ also showed intravenous drug use (IVDU) (p < 0.001) and blood transfusion (p < 0.01), to be statistically significant like our study.

It is noteworthy that in our study many factors like history of used syringes, intra venous drug used, major surgery, sexual contact, tattooing, cesarean section and travel abroad, were not significant in final multiple logistic regression. While factors like travel abroad and tattooing were even not significant at univariate analysis. The possible justification would be female sample population selection; as tattooing and traveling to abroad are not common in females of our society.

It was also interesting to find significant association of female health worker, biopsy and endoscopy with HCV seropositivity. This highlights still poor quality of care provided to the patients with use of unsterilized instruments in tertiary care government facilities. Even our female health workers, main health care providers are not using safe practices. The most important finding of our study was to find dilation and curettage to be statistically significant, even in multiple regression models, along with blood transfusion and dental procedures; of course again it was due to our female sample population. But these three factors all pointing towards inadequate and inefficient quality of care.

There are some limitations of the study. First, who refused to participate in the study may limit the general liability of the sample. Second, it may be difficult to self-report the inherent like injecting drug use etc. Proper counseling including explaining the purpose and objectives of the study, especially by trained medical reactionaries, was used to minimize the above limitations. Third, our findings are limited by the lack of information regarding active HCV infection; as the presence of HCV antibodies only indicates prior exposure. However, since most patients exposed to hepatitis C develop chronic infection,^13^,^14^ HCV antibody testing provides a reasonable estimate of the amount of HCV infection in a population. Finally, the selection of cross sectional study was unable to determine the biologic plausibility between HCV and identified factors.

We observed a significantly high prevalence of HCV in Islamabad 24.6%; even five folds more than in 2004.

This is an alarming sign for an urban area like Islamabad. More so because of the factors identified. The association of these factors, blood transfusion, dental procedure and dilation and curettage, with HCV has proved an inadequate unsafe health practices and quality of care in developing countries. Moreover, previous studies have not shown dilation and curettage to be a significant factor associated with HCV.

Massive health education and promotion should be done in order to control and combat HCV epidemic.
